# Bradykinin Sequestration by *Plasmodium berghei* Infected Erythrocytes Conditions B2R Signaling and Parasite Uptake by Fetal Trophoblasts

**DOI:** 10.3389/fmicb.2018.03106

**Published:** 2018-12-19

**Authors:** Luciana Vieira de Moraes, André Barateiro, Patrícia Marques Sousa, Carlos Penha-Gonçalves

**Affiliations:** Disease Genetics, Instituto Gulbenkian de Ciência, Oeiras, Portugal

**Keywords:** trophoblasts, placental malaria, bradykinin, bradykinin receptor 2, IL-6

## Abstract

*Plasmodium* infection during pregnancy causes placental malfunction reducing fetus sustainability and leading to abortions, stillbirths, low birth weight or premature delivery. Accumulation of infected erythrocytes (IE) in the placenta is a key factor in placental malaria pathogenesis but the role played by fetal trophoblast that contact maternal blood has been neglected. Here we explore the hypothesis that interactions between *Plasmodium*-IE and fetal trophoblast cells impact on vasoactive alterations underlying placental dysfunction. We screened gene expression of key mediators in vasoactive pathways. We found that mRNA of bradykinin receptor 2 (B2R) and nitric oxide synthase (eNOS), as well as levels of bradykinin (BK), were decreased in late gestation placentas of pregnant *Plasmodium berghei*-infected mice. Co-culturing mouse trophoblasts with IE down-regulated B2R transcription and interleukin (IL)-6 secretion in a B2R-signaling dependent manner. IE showed increased levels of surface B2R and enhanced capacity to bind BK. We propose that down-regulation of B2R signaling in the course of IE–trophoblast interactions is due to BK sequestration by IE. In corroboration, levels of BK were lower in infected placentas and the positive correlation of B2R gene expression and fetal weight was disrupted by infection. This indicates that deregulation of the BK-B2R pathway is associated to placental dysfunction provoked by malaria infection. We further found that upon inhibition of B2R signaling, trophoblasts engulf IE to a lesser extent and show reduced production of IL-6. Our data suggest that BK sequestration by *P. berghei* represents a strategy for the parasite to ameliorate the risk of phagocytic capture by down modulating B2R activation.

## Introduction

*Plasmodium* infections during pregnancy are frequently deleterious to the fetus, causing intrauterine growth retardation, stillbirth and abortion [reviewed in (Brabin et al., 2004)]. Current models of placental malaria pathogenesis are focused on linking placental sequestration of infected erythrocytes (IE) with triggering of intra-placental inflammatory events. Fetal trophoblast cells are key components of the maternal-fetal barrier in direct contact with maternal blood and constitute the main fetal cell-type interacting with IE. It seems likely that trophoblast–IE interactions impact on cellular functions that are critical to placental physiology. It has been suggested that nutrient transport across trophoblast is impaired in the infected placenta (Gaccioli and Lager, 2016) and we proposed that iron transport across the placenta is disrupted by malaria infection (Penha-Gonçalves et al., 2014). Epidemiological and experimental evidence converge on the notion that reduced maternal blood supply is a key pathogenesis event in the placental malaria due to imbalance in angiogenesis (Conroy et al., 2009; Silver et al., 2010, 2011) and disturbance of placental microcirculation (de Moraes et al., 2013; Vieira de Moraes and Penha-Gonçalves, 2013). Little is known about the role of trophoblast–IE interactions in mediating these pathogenic effects. It is well established that both angiogenic and vasoregulatory factors are critical for placental invasion and implantation as well as maintenance of normotension in uteroplacental perfusion during pregnancy (Valdes et al., 2009).

Vasodilatory factors have been detected on human trophoblasts, including vascular endothelial growth factor (VEGF) and its receptor VEGFR2 (Tseng et al., 2006), bradykinin receptor 2 (B2R) (Valdés et al., 2001) and endothelial nitric oxide synthase (eNOS) (Corthorn et al., 2006). These factors show altered expression in complicated pregnancies (Schiessl et al., 2005; Corthorn et al., 2006; Tseng et al., 2006). Different lines of evidence support the notion that deregulation of vasoactive mediators contributes to pathology in placental malaria. Angiogenesis deregulation as a consequence of increased levels of complement factor C5a in pregnant women infected with *Plasmodium falciparum* is associated to low birth weight (Conroy et al., 2009). Levels of angiogenic factors such as angiopoetin-1 are reported to be decreased and inversely associated with placental barrier thickness which in turn impacts on materno-fetal nutrient exchange (Ataíde et al., 2015). Adverse pregnancy outcomes are associated with lower concentrations of nitric oxide (NO) precursor, L-arginine, and higher concentrations of endogenous inhibitors of NO biosynthesis in Malawian women with malaria (McDonald et al., 2018).

Bradykinin (BK) is a short-lived nonapeptide mediator generated from high molecular weight kininogen (HMWK) via the action of the plasma enzyme kallikrein. BK in turn binds to B2R – a type 1 G-protein coupled receptor (GPCR) – constitutively expressed in vascular endothelial and smooth muscle cells [reviewed in (Howl and Payne, 2003)]. Binding of BK to B2R induces vasodilation, via release of NO (Drummond and Cocks, 1995) and/or prostaglandins (PGs) (Cherry et al., 1982) and contributes to inflammatory hyperaemia. B2R activation induces interleukin (IL)-6 production in airway smooth muscle cells (Huang et al., 2003). IL-6 is a pleiotropic cytokine with proinflammatory properties and is found to be elevated in patients with symptomatic asthma (Broide et al., 1992). B2R activation-induced IL-6 production has also been described in synovial fibroblast (Lee et al., 2008) and colorectal cancer cells (Wang et al., 2014), suggesting that BK signaling mediates pro-inflammatory responses.

Our previous *in vivo* observations suggested that mouse trophoblasts actively participate in the remodeling of maternal blood spaces, by opening-up or closing these regions in the labyrinthine zone of the placenta (de Moraes et al., 2013). Extending from these observations and literature evidence that trophoblasts express vasoactive factors (Schiessl et al., 2005; Corthorn et al., 2006; Tseng et al., 2006) we investigated if *Plasmodium* infection could affect the expression of vasodilator factors, helping to explain poor pregnancy outcomes in experimental placental malaria (EPM). We screen known key factors of placenta vasoactive pathways and found that B2R and eNOS mRNA transcripts, and BK levels were decreased in the placentas from infected mothers at end-stage pregnancy. This observation led us in turn to investigate the BK-IL-6 axis in the context of IE–trophoblast interactions.

## Materials and Methods

### Animals and Pregnancy Monitoring

All procedures involving laboratory mice were carried in accordance with national (Portaria 1005/92) and European regulations (European Directive 86/609/CEE) on animal experimentation and were approved by the Instituto Gulbenkian de Ciência Ethics Committee and the Direcção-Geral de Veterinária (Official National Entity for regulation of laboratory animals usage). Eight-to-twelve week-old BALB/c female and C57Bl/6 (B6) male mice were obtained from our animal facility at Instituto Gulbenkian de Ciência. Mice were bred and maintained under specific-pathogen free (SPF) conditions. Two BALB/c females were transferred to a cage with one B6 male and removed after detection of vaginal plugs; day of removal was scored as gestational day 1 (G1). Females were weighed immediately after separated from males, and by G12 the gain of approximately 5 g of body weight was confirmative of pregnancy.

### Parasites and Infection

All experiments were conducted with *Plasmodium berghei* ANKA-GFP parasites. IE preparations were obtained from one *in vivo* passage in BALB/c non-pregnant mice, isolated when the percentage of peripheral IE reached approximately 3–5%. Parasitemia was measured by flow cytometry to detect GFP signal. Pregnant mice were infected intravenously on G13 with 10^6^ IE. For *in vitro* experiments we used synchronized IE to obtain schizonts as previously described (Janse et al., 2006). Briefly, parasites were expanded after one passage and animals bled when parasitemia reached 1–3%. Red blood cells were resuspended in RPMI containing 20% fetal bovine serum (FBS) and 0.1% of neomycin and incubated for 18 h in 75 cm^2^ tissue culture flasks, in a total volume of 50 ml. Schizont stage IEs were isolated using MACS 25LS columns (Miltenyi Biotec) according to manufacturer’s instructions (de Moraes et al., 2016).

### Study Design

#### *In vivo* Experiments

Pregnant mice were infected on G13. Mice were euthanized on days 3 (G16), 4 (G17), or 5 (G18) after infection. Fetuses were weighed and placentas collected for immunohistochemistry, gene expression analysis or preparation of placental supernatants.

#### *In vitro* Experiments

Non-infected pregnant mice were euthanized at G18; placentas were collected and processed for trophoblast isolation. Cultured trophoblasts were incubated with synchronized IE (1:1 ratio trophoblasts/IE) or non-IE for 4 or 20 h.

### Sample Collection

#### Placental Supernatants

Placentas were homogenized in 250 μL PBS using a strainer and centrifuged at 16,000 × *g* for 10 min at 4°C. Supernatants were collected and concentrated using Amicon Ultra-0.5 mL centrifugal filters (Merck Millipore). Samples were stored at −80°C until further processing.

#### Serum

Mice were bled by cardiac puncture after euthanasia. Blood was set for 30 min at room temperature, then centrifuged at 1600 × *g* for 10 min to separate serum from cellular and particulate matter. Sera were collected and stored at −80°C until use.

### Gene Expression Analysis and Quantification

Placentas were homogenized and total RNA was extracted using RNeasy Mini Kit (Qiagen) following manufacturers instructions. RNA (1 μg) was converted to cDNA using Transcriptor First-Strand cDNA Synthesis Kit (Roche). *P. berghei* 18S rRNA was amplified with TaqMan-specific primers [forward, 5′-CCGATAACGAACGAGATCTTAACCT-3′; reverse, 5′-CGTCAAAACCAATCTCCCAATAAAGG-3′; probe, 5′-ACTCGCCGCTAATTAG-3′ (FAM/MGB)]. Quantification of mRNA transcripts of the vasoactive receptor genes *Bdkrb2* (Mm00437788) and *Nos3* (Mm00435217_m1) was performed using best-coverage TaqMan Gene Expression Assays (Applied Biosystems) in Real-Time PCR reactions using QuantStudio 7 Flex System (Applied Biosystems). Detected transcript levels were normalized to endogenous control (mouse GAPDH; Applied Biosystems) and relative quantification was obtained by the ΔΔCt method.

### Isolation of Trophoblasts

Placentas from non-infected pregnant mice from G18 were processed for isolation of trophoblasts in procedures adapted from a previously published protocol by (Pennington et al., 2012; Rodrigues-Duarte et al., 2018). Briefly, pregnant mice were euthanized by CO_2_ narcosis and the uterus was carefully removed. Placentas were separated from the feto-placental unit and disrupted in a Petri dish in digestion buffer, consisting of DMEM supplemented with 20 mM HEPES (Gibco), 0.35 g/L sodium bicarbonate (Gibco), 1 mg/mL of collagenase IA (Sigma-Aldrich), and 4 U/μL of DNase. Cells were transferred to 50 mL tubes and incubated in 37°C water bath for 30–40 min with vigorous pipetting every 10 min to digest the disrupted placentas. Placentas from the individual mothers were pooled (approximately 6–8 placentas) and digested in 25 mL of digestion buffer. The digested tissue was passed through a 70 μM pore size strainer and washed with wash buffer (DMEM supplemented with 20 mM HEPES and 0.35 g/L sodium bicarbonate) at 400 × *g* for 10 min at 4°C. Cells were then added to a Percoll (Sigma-Aldrich) gradient as described elsewhere (Nagamatsu et al., 2004). Pelleted cells were resuspended in 4 mL of 25% Percoll, being gently layered on the top of 4 mL of 40% Percoll in a 15 mL tube. These layers were prepared by diluting 90% Percoll in wash buffer previously prepared with PBS 10X. Two milliliter of PBS 1X were added to the top of 25% layer, followed by a centrifugation of 800 × *g* for 20 min at 4°C. Cells at the interface between the two Percoll layers were collected, washed with PBS 1X. Cells were washed, counted and plated in 24-well plate at a density of 2 × 10^5^ cells/well. Cells were cultured in DMEM complete medium containing 10% FBS (Gibco), 1% HEPES (Gibco), 1% sodium pyruvate (Gibco), 1% penicilin and streptomicin (Gibco), 1% non-essential amino acids (BioWhittaker), 1% glutamine, 0.1% β-mercaptoethanol (Gibco), and 0.1% gentamicin (Sigma). Cells were cultured for 1 week to enrich for trophoblasts and washed every 2–3 days to remove non-adherent cells. On the day preceding the experiment, complete medium containing 10% FBS was exchanged for 2% FBS-containing medium. All *in vitro* experiments were conducted with 2% FBS. For *ex-vivo* B2R surface expression, trophoblasts were immediately labeled with fluorescent antibodies and analyzed by flow cytometry (FACS).

### Cell Cultures

Synchronized IE or non-infected erythrocytes (NIE) (2 × 10^5^/well) were added to trophoblasts cultures and incubated for 4 or 20 h at 37°C, 10% CO_2_ in the presence or absence of 1 or 10 μM of bradykinin (Sigma) (BK), 1 μM of B2R antagonist (HOE 140; Sigma). B2R antagonist treatment was performed 15 min before addition of other stimuli. Following the incubation, supernatants were collected and stored at −20°C; wells were washed three times with PBS 1X to discard free/unbound erythrocytes and trophoblasts were incubated with 200 μL accutase (Thermo Fisher Scientific) for 15 min at 37°C. Complete medium (500 μL) was added to block enzyme activity. Cells were collected, washed once in PBS 1X and labeled with anti-B2R and anti-KRT7 fluorescent antibodies. Alternatively, trophoblasts were washed and stored at −20°C or immediately processed for cDNA synthesis using Cells-to-CT kit (Ambion).

### Flow Cytometry

Cultured primary trophoblasts were stained in U-shaped bottom 96-well plates (2–4 × 10^5^ cells/well). Cells were first incubated with 100 μL of Fc Block (2.4G2 ab specific for FcγII/III) diluted in PBS 1X containing 2% FBS and 0.1% sodium azide (NaN_3_) (FACS buffer) for 15 min at 4°C and then washed at 550 × *g* for 1 min at 4°C. Pelleted cells were incubated with 50 μL of the anti-B2R Cy5 antibody (1:100) (Bioss Antibodies) for 30 min at 4°C. Cells were washed twice and resuspended in 100 μL of Fixation/Permeabilization solution (BD Biosciences) for 20 min at 4°C. Fixed cells were washed twice with Perm/Wash solution 1X (BioLegend) to keep them permeable for intracellular staining. After washing, cells were incubated with 50 μL of Perm/Wash 1X containing the anti-KRT7 (Cytokeratin 7) PE antibody (1 μg per 2–4 × 10^5^ cells) (Santa Cruz Biotechnology) for 30 min at 4°C. Finally, cells were washed twice with Perm/Wash 1X and then with FACS buffer prior to cytometry analysis. Cells were resuspended in 100–400 μL of FACS buffer and acquired using the LSR Fortessa X-20 cytometer (BD Biosciences).

### Bradykinin, IL-6 Measurements

Bradykinin levels were evaluated in serum and placental supernatants by competition ELISA (Cloud-Clone Corp.). IL-6 levels were evaluated in culture supernatants by ELISA (Ready-SET-Go!, Affymetrix, eBioscience). All assays were performed according to manufacturer’s instructions.

### Statistical Analysis

Data are presented as mean values ± SD. Unpaired T-tests, Mann Whitney, ANOVA followed by Sidak’s multiple comparison tests for the adjustment of *P* values were performed using the GraphPad Prism 7.0 software. Comparisons between data with probability value of *p* < 0.05 were considered to be significant.

## Results

### Infection Reduces Fetal Weight at G18 and Impinges on Vasodilatory Factor Levels

**FIGURE 1:**
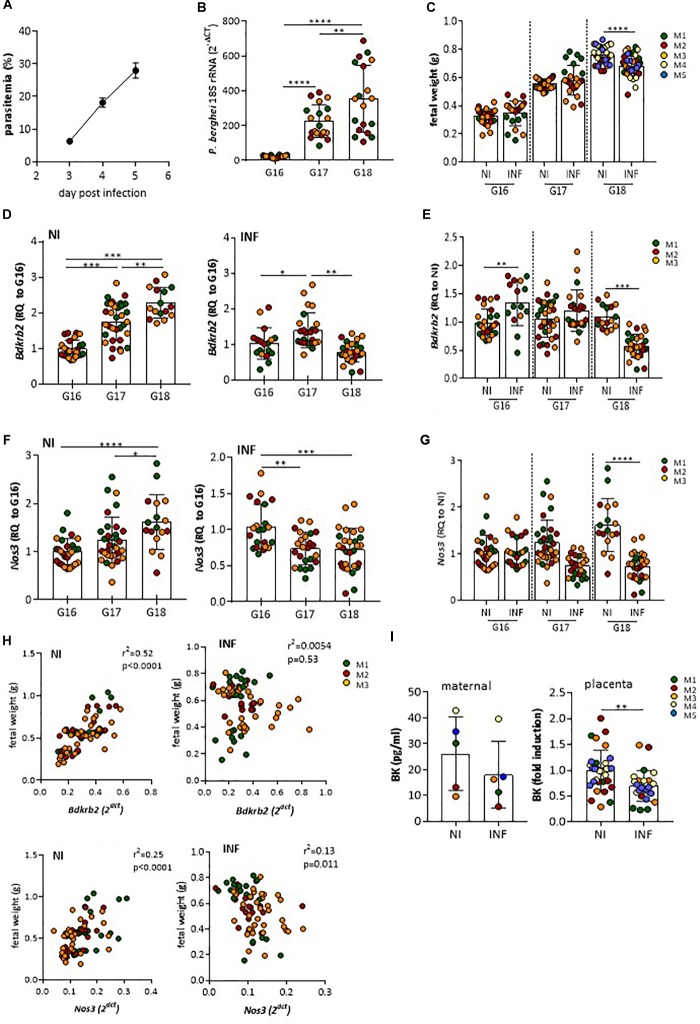
Infection affects mRNA expression of B2R and eNOS genes, BK secretion and associates with low fetal weight. Pregnant mice were infected at G13 with 10^6^ *Plasmodium berghei* IE. **(A)** maternal parasitemia levels, **(B)** placental parasite burden, and **(C)** fetal weight throughout G16–G18 as compared to non-infected (NI); **(D)** kinetics of mRNA expression of *Bdkrb2* in non-infected (NI) and INF placentas [relative quantity (RQ) to G16] and **(E)** in NI vs. INF showing reduced expression of *Bdkrb2* mRNA at G18 in INF placentas; **(F)** kinetics of mRNA expression of *Nos3* in non-infected (NI) and INF placentas [relative quantity (RQ) to G16] and **(G)** in NI vs. INF showing reduced expression of *Nos3* mRNA at G18 in INF placentas; **(H)** correlation between *Bdkrb2* or *Nos3* mRNA expression levels in placentas and correspondent fetal weight from NI and INF mothers (each dot represents one fetal-placental unit from the referred mother (M) at G16-G18). **(I)** Bradykinin levels at G18 in maternal circulation and placental supernatants; each dot represents one placenta; color of placentas matches color of the mother (M); ^∗^*P* < 0.05; ^∗∗^*P* < 0.01; ^∗∗∗^*P* < 0.001; ^∗∗∗∗^*P* < 0.0001 [one-way ANOVA followed by Sidak’s multicomparison test in **B**, **D**, **F**; Unpaired *t* test in **C**, **E**, **G**, **I**; linear correlation (Pearson’s) in **H**].

To ascertain whether vasoactive factors were altered in the course of placental malaria we used a previously described mouse experimental system: Pregnant females were infected with *P. berghei* IE at G13, a gestational stage when maternal blood circulation in the placenta and fetal hemotrophic nutrition are well established (Neres et al., 2008). Maternal parasitemia (Figure 1A), increased steadily from G16 to G18 and placenta parasite burden (Figure 1B) reached high levels by day G17 in line with expectations but we noted that fetal growth retardation, as measured by fetal weight, was only detected at G18 (Figure 1C). This suggested that placental dysfunction impacting on maternal-fetal exchanges is observed by G18. We screened mRNA expression of vasoactive molecules and vasoactive receptors comparing infected and non-infected placentas. We found that mRNA expression levels of genes coding for B2R (*Bdkrb2)* and eNOS (*Nos3*), two molecules involved in vasodilation were deregulated in infected placentas during the G16-G18 time-window. *Bdkrb2* transcription was up-regulated during normal pregnancy but seen to significantly decrease between G17 and G18 in infected placentas (Figures 1D,E). Expression levels of *Nos3* also increased during normal pregnancy, but was found to remain at low levels in infected placentas in the G17–G18 period (Figures 1F,G). The relevance of these findings is highlighted by the positive correlation of *Bdkrb2* mRNA (and to a lesser extent *Nos3* mRNA levels) *w*ith fetal weight in non-infected mothers in the G16–G18 time-window. Such a correlation of observed in the animal cohort with infection (Figure 1H). This suggests that disturbance of B2R signaling during placental infection paralleled with placental dysfunction that affect fetal growth. BK levels were also decreased in placental circulation at G18, but not in maternal blood, compared to normal pregnant mice (Figure 1I). Taken together these data show that *P. berghei* infection during pregnancy affects fetal development in the G17–G18 time window and suggest that infection affects the bradykinin receptor 2 (B2R) signaling pathway.

### Modulation of B2R Expression by BK During Infection

**FIGURE 2:**
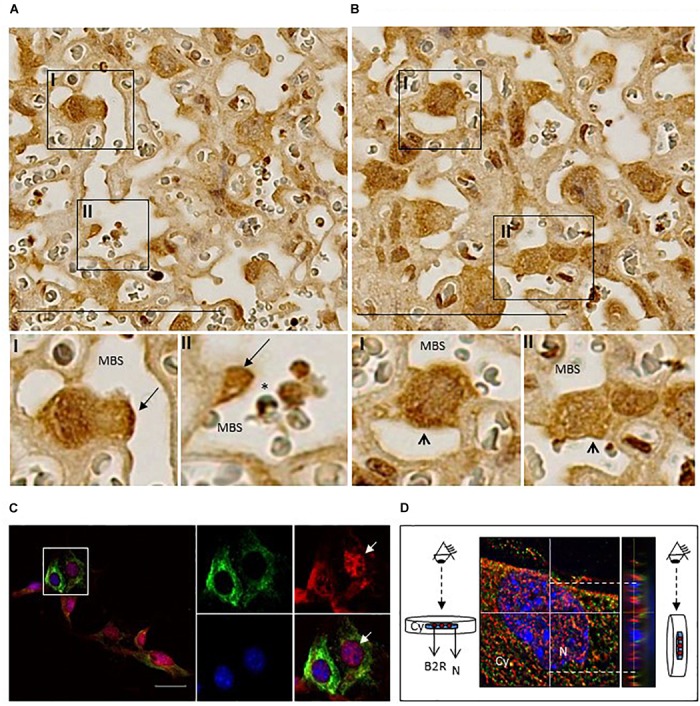
Localization of B2R protein expression in placentas and cultured trophoblasts. **(A)** Immunohistochemistry of infected placentas showing B2R expression in mononuclear trophoblasts protruding to MBS (arrow; I and II) and in **(B)** Coan-Burton bridges (arrowhead; I and II); scale bar: 100 μM; **(C)** Immunofluorescence analysis of cultured trophoblasts from NI placentas; trophoblasts were stained with anti-KRT7-PE (green), anti-B2R-Cy5 (red), and DAPI (blue) and analyzed by fluorescence microscopy (Leica DMRA2); scale bar: 40 μM. The area depicted in the figure is magnified to show individual and merged staining; arrow shows B2R nuclear expression which was confirmed by **(D)** orthogonal views of stack imaging (acquired by Leica High Content Screening microscope) using ImageJ program; Cy, cytoplasm; N, nucleus.

**FIGURE 3:**
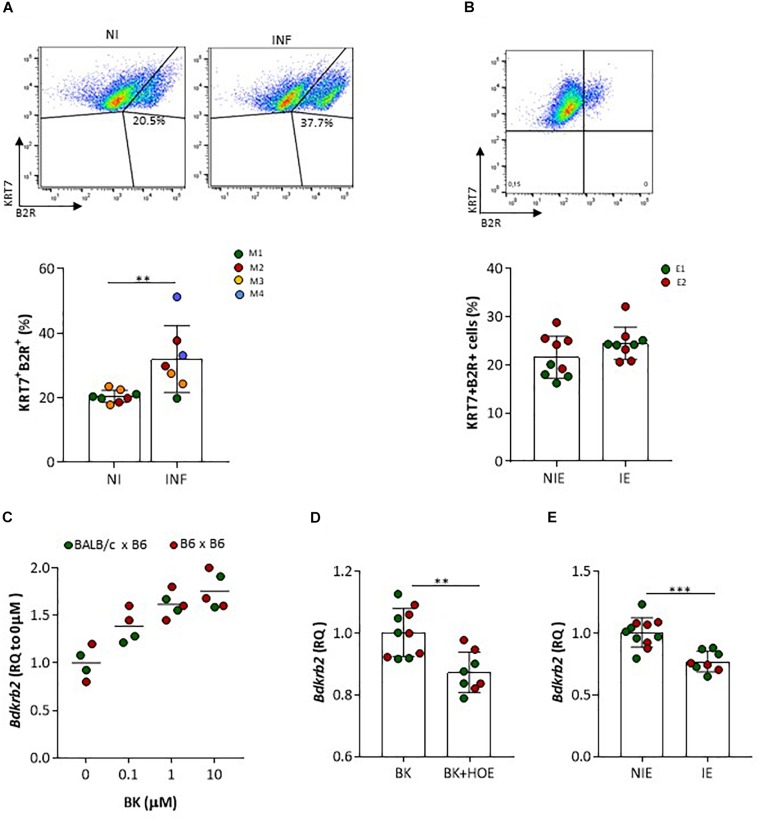
B2R protein and gene expression in trophoblasts. **(A)** Representative FACS plots of placental KRT7^+^B2R^+^ cells isolated from NI or INF pregnant mice; results from 8 NI and 7 INF placentas are compared by Unpaired *t* test; each dot represents one placenta [color of placentas matches color of the mother (M)]; **(B)** FACS plot of B2R^+^ trophoblast (KRT7) isolated from normal placenta cultured for 7 days in the absence of erythrocytes (upper panel); percentage of B2R^+^ trophoblast in serum-free conditions after incubation with non-infected erythrocytes (NIE) or infected erythrocytes (IE) for 4 h; results from 4 to 5 replicates per experiment, from 2 independent experiments (E1 and E2) are compared by Unpaired *t* test (lower panel). Trophoblasts were washed, stained for KRT7 and B2R surface expression and analyzed by FACS. **(C)** *Bdkrb2* mRNA transcription in cultured trophoblasts from allogeneic (BALB/c × B6) or syngeneic (B6 × B6; only for this experiment) pregnancies treated with BK *in vitro* or **(D)** BK in the absence or presence of HOE-140 or **(E)** incubated with NIE or synchronized IE in 2% FBS. Trophoblasts were expanded in culture and incubated with stimuli for 4 h. HOE-140 was added to trophoblasts 15 min before BK, NIE, or IE; ^∗∗^*p* < 0.01, ^∗∗∗^*p* < 0.001 (Unpaired *t* test).

We next assessed by IHC B2R expression in placentas from non-infected pregnant mice at G18 (Figure 2). We observed that B2R was expressed mostly on mononuclear trophoblasts that protrude into maternal blood spaces (MBS) [Figure 2A (I and II)] and in trophoblast bridges (*Coan-Burton* bridges, de Moraes et al., 2013) [Figure 2B (I and II)]. Nuclear staining for B2R was also noted and has previously reported in rat hepatocytes (Savard et al., 2008) and more recently in trophoblasts of the human placenta (Valdés et al., 2016). This cellular expression pattern was confirmed by immunofluorescence staining (Figure 2C) and confocal imaging (Figure 2D) of monolayer cultures of mouse primary trophoblasts, that clearly showed a fraction of trophoblast cells expressing considerable amounts of nuclear B2R. Trophoblast-enriched placental cell suspensions obtained from pools of 4 to 5 placentas at G18 from infected and non-infected pregnant mice were analyzed by flow cytometry. B2R surface protein expression was analyzed among cells expressing KRT7^+^, a cytokeratin expressed in the placenta exclusively by trophoblast (Maldonado-Estrada et al., 2004). The percentage of B2R^+^ trophoblasts was increased in infected placentas (Figure 3A) although surface cellular expression levels were not different from non-infected placentas (data not shown). We then determined whether B2R surface expression in trophoblasts was controlled by BK using an IE–trophoblast interaction assay. We incubated confluent mouse trophoblasts primary cultures with *P. berghei* IE or NIE in serum-free conditions to minimize the influence of serum-derived kininogen precursors in modulation of B2R expression. No differences were observed in the percentage of B2R^+^ trophoblasts (Figure 3B) between non-infected and infected cultures, suggesting that in absence of BK, IE are unable to control B2R surface expression in trophoblasts. Since it has been reported that B2R gene upregulation requires BK binding in NG108-15 neuroglioma cells transfected with B2R promoter (Pesquero et al., 1996), we tested whether BK controls B2R transcription in trophoblasts primary cultures. We found that BK induced B2R gene transcription in trophoblasts in a dose-response manner (Figure 3C). Moreover, trophoblasts treated with the B2R antagonist HOE show reduced BK-induced B2R mRNA transcription (Figure 3D) strongly suggesting that gene regulation is controlled by B2R signaling. Further, we found decrease in B2R gene expression in cultured trophoblasts (in the presence of 2% FBS) exposed to IE *in vitro* (Figure 3E). B2R mRNA levels were slightly but significantly decreased compared to exposure to NIE strongly suggesting that IE impacts on B2R gene expression and possibly on *de novo* synthesis. Together these results suggest that alterations in B2R surface expression, nuclear translocation and gene transcription during infection are controlled through B2R signaling initiated by BK. Nevertheless, it remains unclear how IE control the BK-B2R axis in trophoblasts.

### IE Control Trophoblast B2R Signaling Through Reduction on BK Availability

**FIGURE 4:**
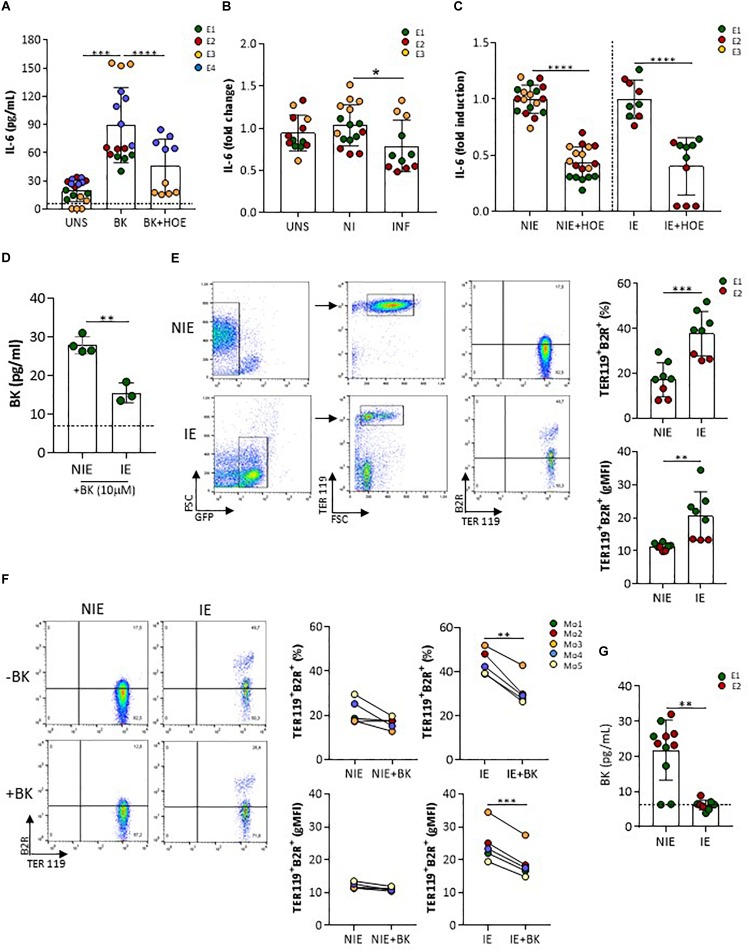
IL-6 production by trophoblasts is impaired by infection and dependent on B2R activation. **(A)** Cultured primary trophoblasts (medium supplemented with 2% FBS) were stimulated with BK (1 μM) or BK in the presence of HOE-140 (10^−6^ M), **(B)** with NIE or IE or left unstimulated, **(C)** or with NIE or IE in the presence of HOE-140 for 4 h. IL-6 was evaluated in the supernatants by ELISA [experimental replicates from 4 **(A)** or 3 (**B** and **C**) independent experiments]; **(D)** BK measurements in the supernatant of NIE or IE suspensions, after incubation with BK (10 μM) in serum-free conditions for 1 h. Supernatants were evaluated for BK by ELISA (data from three biological replicates); **(E)** FACS plot of B2R expression in NIE (gated on GFP^neg^TER119^pos^ erythrocytes) and synchronized *P. berghei* IE (gated on GFP^pos^TER119^pos^ IE) (left panel) and quantification of percentage of B2R^+^ cells and levels of expression (gMFI) of eight biological replicates from two independent experiments (right panel); **(F)** FACS plot of B2R expression in synchronized *P. berghei* IE and NIE incubated for 1 h in serum-free medium and in the presence (+) or absence (–) of BK [gating strategy as described in **(F)** (left panel)] and quantification of percentage of TER119^+^B2R^+^ cells and levels of expression (gMFI) of TER119^+^B2R^+^ erythrocytes (five biological replicates; Mo, mouse). IE were separated from NIE using MACS LS columns and both fractions – infected and non-infected erythrocytes – were stained with anti-B2R antibody. FACS analysis was performed in GFP^+^ and GFP^−^ populations. **(G)** BK levels in the supernatant of a 4-h trophoblast-IE and trophoblast-NIE co-culture (supplemented with 10% FBS). BK was evaluated in the ELISA [two independent experiments (E1 and E2)]. ^∗^*P* < 0.05, ^∗∗^*P* < 0.01, ^∗∗∗^*P* < 0.001, ^∗∗∗∗^*P* > 0.0001 (one-way ANOVA followed by Sidak’s multicomparison test in **A**,**B**; Unpaired *t* test in **C–E**,**G**; Paired *t* test in **F**); dashed line in **D**,**G** refers to limit of detection level of BK.

Next, we identified whether B2R signaling on trophoblasts were altered upon interaction with IE *in vitro*. In vascular endothelial cells, activation of B2R by BK results in calcium (Ca^2+^)-dependent eNOS activation and production of NO (Venema, 2002). We did not detect neither intracellular Ca^2+^ mobilization nor nitrite accumulation in trophoblast cultures after BK stimulation (data not shown). *Nos3* mRNA expression showed late amplification suggesting that in isolated and cultured trophoblasts this gene might be poorly expressed (data not shown). On the other hand, IL-6 has been described as a downstream factor of B2R activation in a renal tubular cell line (Ramani et al., 2016) and in human decidua-derived cells (Rehbock et al., 1997). We found that IL-6 production was increased in presence of BK and was inhibited in presence of HOE-140, a B2R-signaling specific antagonist (Figure 4A). Interestingly, levels of IL-6 were lower in IE-exposed trophoblast cultures compared to NIE-exposed cultures (medium supplemented with 2% FBS) (Figure 4B) and showed a two-fold reduction in the presence of B2R antagonist, suggesting that down-regulation of IL-6 production upon interaction with IE operates through inhibiting B2R signaling (Figure 4C). BK levels in the supernatant of trophoblast-IE co-cultures were also decreased compared to trophoblast-NIE cultures (Figure 4G). In this case we performed co-cultures in the presence of 10% FBS that allows the detection of consistent amounts of BK. Therefore, we hypothesized that the decrease of IL-6 production in infected trophoblast cultures could be a result of BK sequestration, compromising B2R signaling. To test this possibility, we incubated IE or NIE with BK (10 μM) in serum-free medium for 1 h and evaluated BK levels in the supernatant. BK free levels were found to be lower when incubating with IE as compared to NIE (Figure 4D), suggesting that IE reduce BK availability. BK levels were also decreased in infected placentas at G18, but not in maternal blood, indicating that accumulation of IE in the placenta reduces local BK availability (Figure 1I) corroborating the previous observations. In summary, these findings suggest that IE reduce BK availability impairing B2R signaling.

### B2R Expression in IE Reduces BK Availability

Given that B2R has been reported to be expressed on the erythrocytic membrane (Silva et al., 2016), we compared B2R surface expression in IE and NIE. Our data show that IE have increased percentage of B2R positive cells and display a population with high B2R surface expression (Figure 4E), strongly suggesting that IE have increase capacity to sequester BK. It has been proposed that B2R exposure to agonists leads to internalization of the BK-B2R complex in cell lines (de Weerd and Leeb-Lundberg, 1997; Haasemann et al., 1998). We evaluated B2R expression in IE and NIE after incubation with 10 μM of BK for 1 h. Both the percentage of B2R^+^ IE and the levels of B2R surface expression were significantly decreased when IE were incubated with BK, whilst only a mild effect was observed in NIE (Figure 4F). We suggest that the decrease of B2R expression after BK exposure in IE cultures is due to receptor internalization, as described in other cell types. Together, these results strongly suggest that during IE–trophoblasts interaction IE have increased ability to sequester free BK, reducing BK availability which results in turn in decreasing B2R signaling in trophoblasts (and lower production of IL-6).

### Trophoblast B2R Signaling Is Involved in IE Engulfment

**FIGURE 5:**
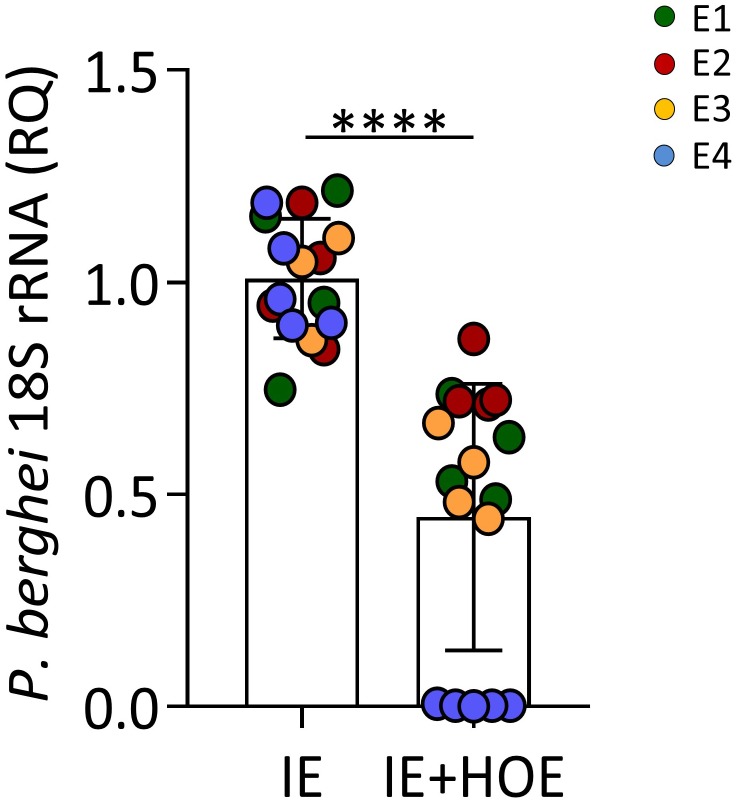
B2R activation is associated with IE phagocytosis. *P. berghei* 18S rRNA expression in 4-h trophoblasts culture infected with synchronized IE in the presence or absence of HOE-140, showing reduced parasitic load in the absence of B2R signaling; ^∗∗∗∗^*P* < 0.0001 (Unpaired *t* test).

It has been shown that B2R activation in human trophoblast cell lines induces migration and invasion (Erices et al., 2011); we therefore evaluated the impact of B2R signaling on IE engulfment – a process associated with trophoblast motility. Trophoblast cultures were incubated in the presence or absence of HOE-140 prior exposure to IE and the parasite engulfment was evaluated by qPCR. Results reveal decreased amounts ofPb18S rRNA in cultures treated with B2R antagonist (Figure 5), suggesting that B2R signaling contributes to effective engulfment of IE by trophoblast cells. Together these results unveil that up-regulation of B2R on the IE membrane favors BK sequestration reducing BK availability and impairing trophoblast responses to IE.

## Discussion

**FIGURE 6:**
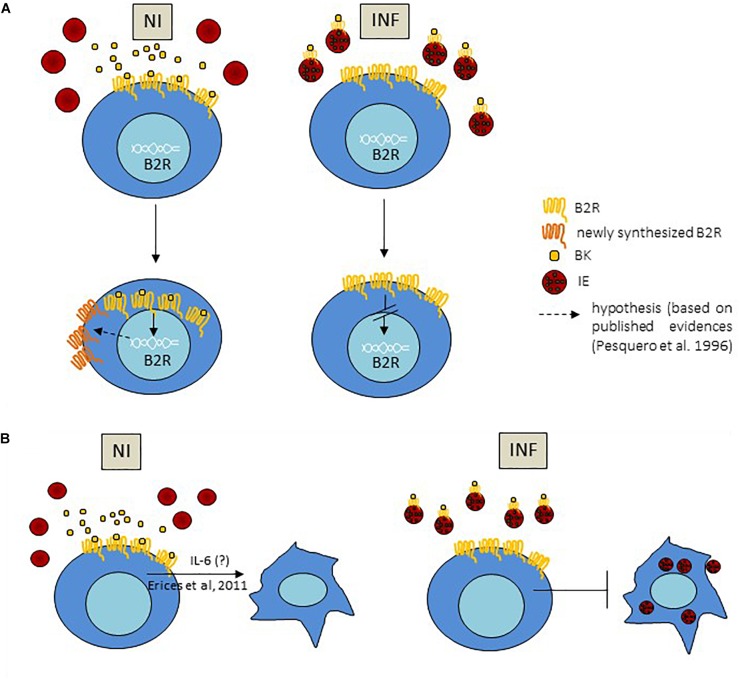
Proposed mechanism for BK sequestration by IE impacting on B2R expression and IE engulfment by trophoblast; **(A)** BK sequestration by IE, via interaction of BK with B2R on the IE membrane, impact on B2R gene expression in trophoblasts leading to decrease in mRNA transcription in these cells; increase of B2R^+^ trophoblasts in infected conditions compared to non-infected reflects less internalization of the complex BK-B2R due to lower BK availability. **(B)** B2R activation induces trophoblast invasion and migration phenotypes (Erices et al., 2011) and we hypothesize that this effect is mediated by IL-6; secretion of IL-6 downstream of B2R signaling is decreased in the presence of IE which may impact on trophoblast movement and IE phagocytosis by trophoblasts.

This study uncovers a mechanism of BK sequestration by IE, conditioning B2R signaling and IE engulfment by trophoblasts. We offer an explanation for the disturbances of B2R expression observed in malaria during pregnancy. IE showed increased expression of surface B2R and seem to have an increased capacity to capture BK. In the context of *in vitro* trophoblast–IE interactions we found that B2R signaling is down-modulated suggesting that IE reduce BK availability and consequently BK-induced B2R activation in trophoblasts. Interestingly, decreased B2R signaling in trophoblasts results in reduced efficiency of IE engulfment (summarized in Figure 6). These *in vitro* observations are paralleled by the analysis of placentas from infected mice. We found that both BK availability and B2R gene expression are reduced in the infected placenta. Further, we found that the correlation of B2R expression with fetal weight in late pregnancy seen in healthy, uninfected animals, was disrupted by infection. This suggests that pathological effects of placental IE accumulation include the dysregulation of B2R signaling in trophoblasts, leading to placental dysfunction and restricting fetal growth.

The presence of B2R on the erythrocytic membrane has been recently described (Silva et al., 2016). The authors propose a synergistic effect between B2R and MAS receptor activation reducing downstream PKA activity and inhibiting *P. falciparum* merozoite invasion. Because *P. berghei* parasites do not invade erythrocytes *in vitro*, testing of this hypothesis is limited in the context of our experimental system. Nevertheless, we show that, once erythrocytes are infected, B2R expression in the erythrocyte membrane is up-regulated, increasing the capacity to bind and deprive BK from the medium. The observation that B2R expression in IE is down-regulated after exposure to BK, suggests a mechanism that is not operating in NIE. We speculate that this mechanism is operating in IE and therefore would explain decrease in B2R surface protein expression because exposure of BK to B2R has been shown to cause downregulation of B2R surface levels (Roberts and Gullick, 1990; Wolsing and Rosenbaum, 1991). Although it is unclear if BK sequestration by IE affects parasite growth, it is plausible that a decrease in local BK availability deprives surrounding host placental cells from B2R signaling. NIE expression of B2R appears to have a protective role against parasite invasion (Silva et al., 2016); here we propose that increased B2R expression on the IE membrane conditions the responses of trophoblasts in the course of infection.

Using primary trophoblast cultures, we found that BK increased B2R transcription in a dose-response manner, an effect that was inhibited by B2R antagonism, suggesting that B2R signaling in trophoblasts is governed by a feed forward mechanism. Increase in transcriptional activity of the B2R promoter after BK treatment has already been previously reported (Pesquero et al., 1996). On the other hand, exposure of B2R to BK has been shown to cause reduction in receptor-ligand affinity (desensitization), downregulation of B2R surface levels (Roberts and Gullick, 1990; Wolsing and Rosenbaum, 1991) and appearance of low affinity receptors (Roberts and Gullick, 1990). This has led to the proposal that BK-induced B2R transcription may represent a long-term regulation pathway, leading to an efficient replacement of low affinity receptors by newly synthetized molecules (Pesquero et al., 1996). Our data show that infected placentas had a higher percentage of B2R+ trophoblast compared to NI placentas which did not correlate to B2R gene expression: *Bdkrb2* levels were significantly decreased in INF compared to NI placentas. Our hypothesis/explanation is that decreased B2R transcription is a consequence of low BK availability due to BK sequestration by IE. This means that B2R would be less internalized reflecting on a higher B2R expression on the trophoblast membrane (Figure 6A). Our trophoblast co-cultures corroborate these observations showing that in the presence of IE Bdkrb2 levels were decreased but B2R surface expression was not affected. We observed reduced B2R transcription and an increase in B2R^+^ trophoblasts in infected placentas. which might be explained by low BK availability. Nevertheless, the physiological relevance of B2R signaling in trophoblasts remains an open theme.

We found that IL-6 production in primary trophoblasts cultures is induced by BK and inhibited by B2R antagonism, indicating that IL-6 is a bona-fide readout of B2R signaling in trophoblasts. Interestingly, HOE-140 treatment reduced significantly the amount of IL-6 in non-stimulated cultures implying that BK precursors, which are present in the culture medium, were derived from FBS. Accordingly, IL-6 was undetectable in non-stimulated or in NIE and IE-stimulated cultures in serum-free conditions (not shown), further indicating that B2R signaling is required for IL-6 production in these experimental conditions. Furthermore, it has been reported that expression of kallikrein by the HTR-8/SVneo trophoblast cell line was only detected with media supplemented with FBS, strongly suggesting that endogenous bradykinin derives from kallikrein acting on serum-derived kininogen (Erices et al., 2011). Our findings additionally raise the possibility that kallikrein produced by trophoblasts indirectly controls BK availability and B2R signaling providing an endogenous feed-forward mechanism for physiological control of IL-6 production. Such a mechanism could be impaired when BK availability is altered by exogenous factors such as IE, leading to alterations on B2R downstream effects. Discerning whether BK sequestration by IE *in vivo* has an effect on decreasing IL-6 production by trophoblast would require genetic systems that discriminate between maternal and fetal-derived IL-6 secretion in the placenta.

Interleukin-6 has been shown to be downstream of BK-B2R activation in airway smooth muscle cells (Huang et al., 2003), in synovial fibroblasts (Lee et al., 2008) where it is involved in inflammatory processes, and in colorectal cancer cells promoting cell invasion and migration (Wang et al., 2014). In vascular smooth muscle cells, IL-6 stimulated migration by inducing actin polymerization and tyrosine phosphorylation of focal adhesion-associated cytoskeleton proteins (Wang and Newman, 2003). Several lines of evidence support the possibility that IL-6 production by trophoblasts impacts directly (Jovanović and Vićovac, 2009; Dubinsky et al., 2010) or indirectly (Champion et al., 2012) on trophoblast mobility opening the possibility that the BK-B2R-IL-6 axis is controlling trophoblast motility in the hemochorial placenta.

We show that IL-6 production by trophoblasts is reduced in the presence of IE and is dependent on B2R signaling. It has been proposed that control of B2R signaling governs invasive and migratory trophoblasts properties (Erices et al., 2011) associated with increased filopodia formation. Our finding that inhibition of B2R signal leads to decrease of IE engulfment by trophoblasts might be explained by impairments in trophoblast motility as proposed by (Erices et al., 2011) and may be related to mechanisms of BK-induced phagocytosis in macrophages and polymorphonuclear cells (Stoika et al., 2002). In addition, other physiological roles of IL-6 in the placenta, such as secretion of human chorionic gonadotrophin (hCG) hormone (Nishino et al., 1990) and increase in placental nutrient transport (Jones et al., 2009) could be affected by decreased B2R signaling level in trophoblast provoked by intra-placental IE accumulation.

The unexpected finding that B2R expression is increased in the IE membrane, probably enhancing their BK binding capacity, raises the possibility that in context of placental malaria pathogenesis it represents a mechanism that actively reduces BK availability in maternal blood spaces where IE accumulate. We found that placentas of infected animals had lower levels of BK and reduced transcription of the *Bdkrb2* gene. Interestingly, we found that *Bdkrb2* and *Nos3* expression positively correlated with fetal weight in non-infected placentas. No such correlation was observed in infected placenta, suggesting that these vasoregulatory systems take part on placental dysfunction imposed by infection. Although B2R protein is expressed in trophoblasts, stimulation with BK did not yield detectable intracellular calcium mobilization and nitrite in supernatants (data not shown). *Nos3* mRNA in cultured trophoblasts was expressed at very low levels (data not shown). These results suggest that B2R signaling in trophoblasts is not directly controlling NO production but leave open the possibility that B2R signaling in trophoblasts may impact NO biosynthesis in fetal endothelial cells. With these findings we speculate that accumulation of IE in the placenta is an effective mean to induce local deprivation of B2R signal with implications on placental vasoregulation and on the engulfment and elimination of IE. In an elegant study employing mutant *Leishmania major* promastigotes that lack serine peptidase inhibitors (ISP) and consequently do not act in releasing BK from kininogen bound on the surface of macrophages, strongly suggest that B1R/B2R activation is suppressed due to inhibition of kinin-releasing serine protease activity operating as a parasite mechanism to reduce BK availability that in turn decreases pro-inflammatory responses to *L. major* infection (Svensjö et al., 2014). Thus, we propose BK sequestration by IE has a dual role in placental malaria. On the one hand it works as a parasite strategy to escape IE phagocytosis (Figure 6B); on the other hand, contributes to placental dysfunction and poor pregnancy outcomes in infected mothers.

## Author Contributions

LdM and AB designed and performed the experiments. LdM wrote the paper. PS performed the experiments. CP-G designed the experiments and wrote the paper.

## Conflict of Interest Statement

The authors declare that the research was conducted in the absence of any commercial or financial relationships that could be construed as a potential conflict of interest.
